# Overexpression of type VI collagen in neoplastic lung tissues

**DOI:** 10.3892/or.2014.3438

**Published:** 2014-08-22

**Authors:** LARRY VOILES, DAVID E. LEWIS, LING HAN, IVAN P. LUPOV, TSANG-LONG LIN, MICHAEL J. ROBERTSON, IRINA PETRACHE, HUA-CHEN CHANG

**Affiliations:** 1 Department of Biology, Indiana University-Purdue University Indianapolis School of Science, Indianapolis, IN, USA; 2 Department of Comparative Pathobiology, Animal Disease Diagnostic Laboratory, Purdue University College of Veterinary Medicine, West Lafayette, IN, USA; 3 The Bone Marrow and Stem Cell Transplantation Program, Lymphoma Program and the Division of Hematology/Oncology, Department of Medicine, Indiana University School of Medicine, Indianapolis, IN, USA; 4 Division of Pulmonary, Allergy, Critical Care, Occupational and Sleep Medicine, Department of Medicine, Indiana University School of Medicine and the ‘Richard L. Roudebush’ VA Medical Center, Indianapolis, IN, USA

**Keywords:** type VI collagen, monocyte, lung epithelium, IL-23, FAK, ERK1/2, lung fibrosis, emphysema, lung cancer

## Abstract

Type VI collagen (COL6), an extracellular matrix protein, is important in maintaining the integrity of lung tissue. An increase in COL6 mRNA and protein deposition was found in the lungs of patients with pulmonary fibrosis, a chronic inflammatory condition with a strong association with lung cancer. In the present study, we demonstrated overexpression of COL6 in the lungs of non-small cell lung cancers. We hypothesized that excessive COL6 in the lung interstitium may exert stimulatory effects on the adjacent cells. *In vitro* stimulation of monocytes with COL6 resulted in the production of IL-23, which may promote tumor development in an environment of IL-23-mediated lung inflammation, where tissue modeling occurs concurrently with excessive COL6 production. In addition, COL6 was capable of stimulating signaling pathways that activate focal adhesion kinase and extracellular signal-regulated kinase 1/2 in lung epithelial cells, which may also facilitate the development of lung neoplasms. Taken together, our data suggest the potential role of COL6 in promoting lung neoplasia in diseased lungs where COL6 is overexpressed.

## Introduction

Extracellular matrix (ECM) proteins provide structural support for lung cells involved in gas exchange and may also actively regulate various aspects of lung function in both homeostatic and pathological conditions. Persistent exposure to cigarette smoke or airborne pollutants leads to the recruitment and activation of monocytes and alveolar macrophages (1,2). These, in turn release proteases that destroy ECM components in the alveolar septa, resulting in the development of emphysema, a common type of chronic obstructive pulmonary disease (3). On the other hand, increased degradation of ECM may promote aberrant remodeling of the connective tissues with resultant lung fibrosis. Lung fibrosis and emphysema may coexist and indeed, expression of a large number of ECM-related genes are upregulated in the lungs of smokers with severe emphysema (4,5). Among increased ECM proteins, type VI collagen (COL6) is deposited early in lung fibrosis (6). Both emphysema and lung fibrosis have a strong association with the occurrence of lung cancers (7–9). However, the expression patterns of COL6 in human lung cancers are largely unknown.

Collagen, an ECM protein, is important in maintaining the integrity of the lung tissues (10). The pulmonary interstitial compartment provides the scaffold and connection to the alveolar network via the contents of matrix proteins. Although COL6 is not the major structural element of the lung, it is present in the interstitium of the lung (10), as fine microfibrils with 3–10 nm in diameter that exhibit a double-beaded period of ~100 nm (11). COL6 consists of monomers containing at least 3 major polypeptides, α1, α2 and α3. The monomers aggregate intracellularly into dimers and then tetramers (12). Three additional polypeptides (α4, α5 and α6) have been identified to have high homology to the α3 chain (13,14).

The multi-domain structure of COL6 allows its interaction with components of matrix, including biglycan, decorin (15,16), hyaluronic acid (17), heparin sulphate (18) and type I (COL1) and type IV (COL4) collagen (6,19,20). Such interaction can stabilize ECM architecture, which is important in maintaining the integrity of tissues in the lung (10). In addition to the matrix-interaction, COL6 binds to integrins (21) of various cell types, initiating the matrix-cell interaction and affecting signaling pathways that regulate cellular functions, such as proliferation (22), migration (23), differentiation (24) and survival (25). COL6 has potent growth-stimulatory effects, which is associated with aggressive tumor growth (22). Increased expression of COL6 has also been positively correlated with tumorigenesis (26–29).

In this report, we revealed increased levels of COL6 in the lung tissues of patients with non-small cell lung cancers. Since COL6 is located in the pulmonary interstitial compartment where it encounters various cell types, including infiltrated monocytes and epithelial cells, we hypothesized that excessive COL6 may have effects on its surrounding cells. The objective of this study was to understand the role of excessive COL6 in the lung tissues with aforementioned lung diseases. Our results demonstrated the stimulatory properties of COL6 on various cell types, which in turn may have impacts on the development of lung neoplasia from emphysematous or fibrotic lungs where COL6 is upregulated.

## Materials and methods

### Collagens, antibodies and other reagents

Human collagen type IV (COL4) and VI (COL6) purified chromatographically and immunologically from human placenta were obtained from Rockland Immunochemicals Inc. (Gilbertsville, PA, USA ). Human anti-COL6A1 polyclonal antibody for immunohistochemical staining was obtained from Santa Cruz Biotechnology (Santa Cruz, CA, USA ). Lipopolysaccharide (LPS from *Escherichia coli* 0111:B4) was from Sigma-Aldrich (St. Louis, MO, USA).

### Cell lines, human blood samples and primary cells

The human TH P-1 monocytic cell line (AT CC; American Type Culture Collection, Manassas, VA, USA ; provided by Dr Michael Klemsz from Indiana University School of Medicine) was cultured in complete RPMI-1640 medium. Human blood samples of healthy donors were obtained from the Indiana Blood Center. Peripheral blood mononuclear cells (PBMCs) were separated using Ficoll centrifugation and aliquots of PBMCs were cryopreserved in liquid nitrogen. Human primary monocytes were isolated from PBMCs using CD14 magnetic beads with ~93% of purity (Miltenyi Biotec Inc., Auburn, CA, USA ). Human bronchial epithelial cells (HBEpCs) derived from the surface epithelium of normal human bronchi were obtained from Cell Applications (San Diego, CA, USA ) and were cultured in Bronchial/Tracheal Epithelial Cell Growth Medium provided by the same supplier.

### Gene expression in normal and neoplastic lung tissues

TissueScan Lung Cancer Tissue qPCR Panel II containing 48 tissues covering 4 disease stages (IA, IB, IIA, IIB, IIIA, IIIB and IV) of NS CLC and normal controls were obtained from OriGene Technologies (Rockville, MD, USA). The gene expression of *COL6A1* and *IL23A* was analyzed using the TaqMan assay primers with *ACTB* (β-actin) as endogenous control in the ABI 7300 system (Applied Biosystems by Life Technologies, Carlsbad, CA, USA ).

### Analysis of COL6 protein in human lung tissues using immunohistochemistry staining

Lung disease tissue array slides (LUD481 and LUC962) containing normal controls, adenocarcinoma and squamous cell carcinoma were obtained from US Biomax (Rockville, MD, USA ). The levels of COL6 protein were analyzed using immunohistochemical staining with an anti-COL6A1 polyclonal antibody (Santa Cruz Biotechnology).

### Cytokine production by monocytes

Monocytes (2–4×10^6^ cells/ml) freshly purified from normal PBMCs were treated with medium only, LPS (1 μg/ml) and COL6 at the concentrations of 10 or 30 μg/ml. Eight hours following treatment, the supernatants were collected and the cell pellets were resuspended in TRIzol reagents (Invitrogen, Carlsbad, CA, USA ) for total RNA extraction, first-strand cDNA synthesis (Invitrogen) followed by real-time qPCR with TaqMan assay primers for *IL1B* (IL-1β), *IL6* (IL-6), *TNFA* (TNFα), *IL23A* (p19) and *IL12B* (p40). The levels of cytokine secretion in the supernatants, including IL-23 and TNFα were measured using ELISA as previously described (30).

### Analysis of focal adhension kinase (FAK) and extracellular signal-regulated kinase (ERK) activation following stimulation

TH P-1 or HBEpC cells were stimulated as indicated at 37°C in a 5% CO_2_ incubator for 1 h. Cells were lysed using the RIPA protein lysis buffer consisting of 10% glycerol, 1% Igepal, 50 mM Tris-pH 7.4, 150 mM NaCl, 1 mM EDTA- pH 8.0, 1% Na-deoxycholate, 0.1% SDS and protease inhibitors (31). Activation of FAK was evaluated using western blot analysis with antibodies against phospho-FAK (P-FAK) at Tyr 397 (Y397) and Y925 followed by total FAK (Cell Signaling Technology, Danvers, MA, USA). Activation of ERK1/2 was evaluated using western blot analysis with phospho-P44/42 ERK1/2 (Thr202/Tyr204) followed by total ERK antibodies (Cell Signaling Technology). The band intensity was determined using Image J program (NIH) and the ratios were calculated as the intensity of phosphorylated proteins divided by the intensity of total proteins.

### Statistical analysis

PAS W Statistics (IBM-SPSS, Chicago, IL, USA) was used to analyze the data. P-value was determined with an independent Student’s t-test.

## Results

### COL6 expression is upregulated in neoplastic lung tissues

**Figure 1:**
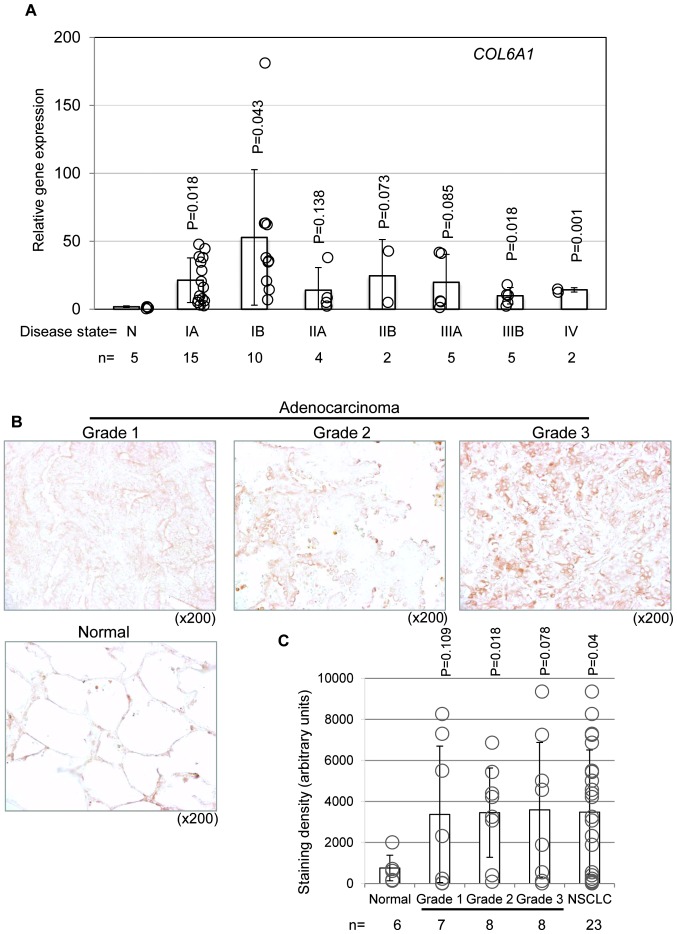
Expression of COL6 in normal and neoplastic lung tissues. (A) *COL6A1* gene expression in human lung tissues. Tissue Scan Lung Cancer Tissue qPCR Panel II containing 48 tissues covering 4 disease stages (IA, IB, IIA, IIB, IIIA, IIIB and IV) of NS CLC and normal tissues (N) was used. The gene expression level of *COL6A1* was analyzed using TaqMan assay primers with *ACTB* (β-actin) as endogenous control. Data are presented as mean ± SD in a bar graph from each group. Open circle (○) indicates the expression level of individual tissue from the group. P-value is compared to the normal tissues. (B) Immunohistochemical staining of COL6 protein from human lung tissues. Lung disease tissue array slides (LUD481 and LUC962) containing normal controls and NSCLC (grade 1–3) were used. The levels of COL6 protein in the slides were analyzed using immunohistochemical staining with anti-COL6A1 polyclonal antibody. Representative image of adenocarcinoma (grade 1–3) and normal lung at higher magnification (x200) is shown from slide LUD481. (C) Intensity of COL6A1 staining in the whole tissues in the slides was analyzed using Image J program and presented as the mean intensity ± SD from normal (n=6) and NS CLC (n=23; total grade 1–3), including grade 1 (n=7; 3 adenocarcinoma and 4 squamous cell carcinoma), grade 2 (n=8; 3 adenocarcinoma and 5 squamous cell carcinoma), grade 3 (n=8; 3 adenocarcinoma and 5 squamous cell carcinoma). P-value is compared to the normal tissues. COL6, type VI collagen; NS CLC, non-small cell lung cancer.

In this study, we evaluated the levels of COL6 in neoplastic lung tissues. The gene expression of *COL6A1* was significantly higher in lung tissues with NSCLC at various stages, as compared to that from normal controls (Fig. 1A). In addition, the levels of COL6 protein were elevated in lung tissues from NS CLC patients with adenocarcinoma and squamous cell carcinoma (Fig. 1B and C). Positive staining for COL6A1 was more intense in the cytoplasm of stromal cells and in the immediately adjacent extracellular space. Uninvolved healthy lung tissue sections displayed the normal structure of alveoli, whereas lung adenocarcinoma sections exhibited various stages of neoplastic differentiation with loss of normal alveolar structures (Fig. 1B). A marked staining of COL6 was noted in 65% (15/23) of the lung tissues from the NS CLC patients analyzed (Fig. 1C). We found no significant difference in COL6 staining from the lung tissues among the three different grades (grade 1–3) of lung cancer (Fig. 1B and C).

### COL6 stimulates monocytes to produce IL-23

During lung inflammation, lung epithelial cells are activated upon exposure to irritants and produce chemokines that attract immune cells from the periphery to the lungs. Among these chemokines, CCL2 attracts CCR2-expressing monocytes (32). Given the fact that elevation of COL6 is present in the pulmonary interstitium, an area shared with recruited monocytes during lung inflammation, we first tested the hypothesis that excessive COL6 has stimulatory effects on monocytes. To investigate whether monocytes respond to COL6, human primary monocytes were purified from PBMCs of normal control donors using CD14^+^ magnetic microbeads with a purity of ~93%. Isolated monocytes were stimulated with different concentrations of COL6 followed by analysis of cytokine production.

**Figure 2:**
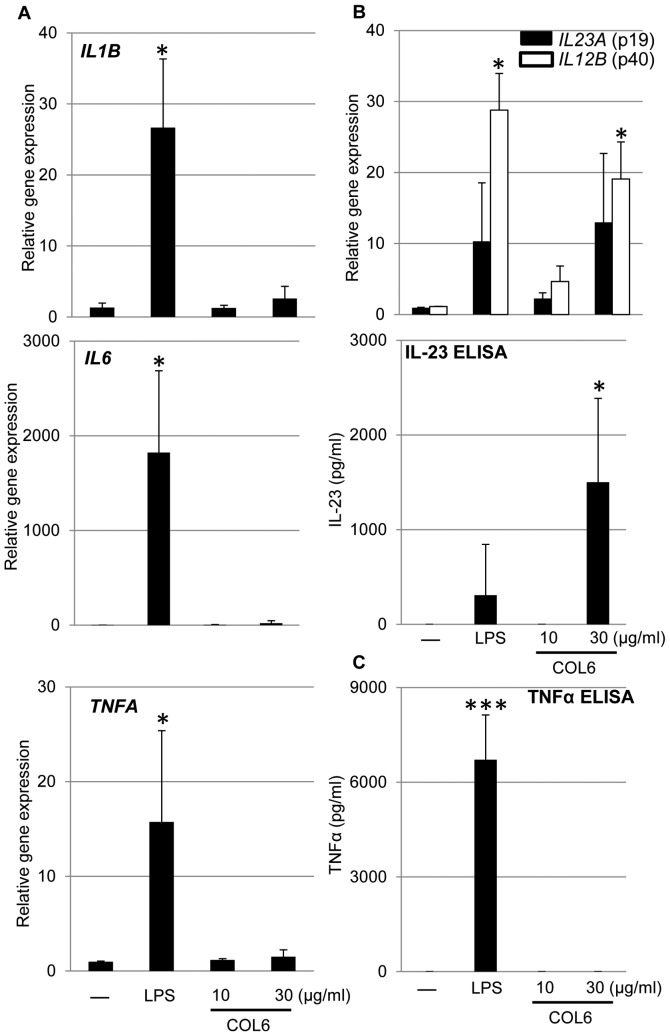
COL6 stimulates human monocytes to produce IL-23. Human primary monocytes were isolated from peripheral blood mononuclear cells (PBMCs) of normal control donors using CD14 magnetic microbeads. Isolated monocytes were stimulated with medium only (−), lipopolysaccharide (LPS, 1 μg/ml) and different concentrations of type VI collagen (COL6) for 8 h. The cell pellets collected following 8 h of stimulation were analyzed for gene expression using qPCR with TaqMan Assay primers for *IL1B*, *IL6*, *TNFA* (A), *IL23A* and *IL12B* (B, upper panel). The levels of IL-23 (p19+p40) (B, lower panel) and TN Fα (C) in the supernatants were determined using ELISA. Results are presented as mean ± SD averaged from 3 different donors. ^*^P≤0.05, ^***^P≤0.001, relative to cells cultured in medium (−).

Human monocytes express toll-like receptor 4 that responds to LPS stimulation, resulting in induction of pro-inflammatory cytokines (33). LPS was therefore used as a positive control in the *in vitro* study. As expected, LPS stimulation in human monocytes resulted in upregulation of pro-inflammatory cytokines such as *IL1B*, *IL6* and *TNFA* (Fig. 2A). We found that COL6 stimulation had no significant effects on expression of *IL1B*, *IL6* and *TNFA* (Fig. 2A). However, increased gene expression of each subunit (*IL23A* for p19 and *IL12B* for p40) of IL-23 was noted following LPS or COL6 stimulation in the monocytes (Fig. 2B, upper panel). In addition, production of IL-23 was dependent on the dose of COL6 as no detectable level of secreted IL-23 was noticed using a lower concentration of COL6 (Fig. 2B, lower panel). Similar to *TNFA* gene expression, secreted TNFα was only detected following stimulation with LPS, but not with medium or COL6 (Fig. 2C).

### IL23A expression is upregulated in neoplastic lung tissues

**Figure 3:**
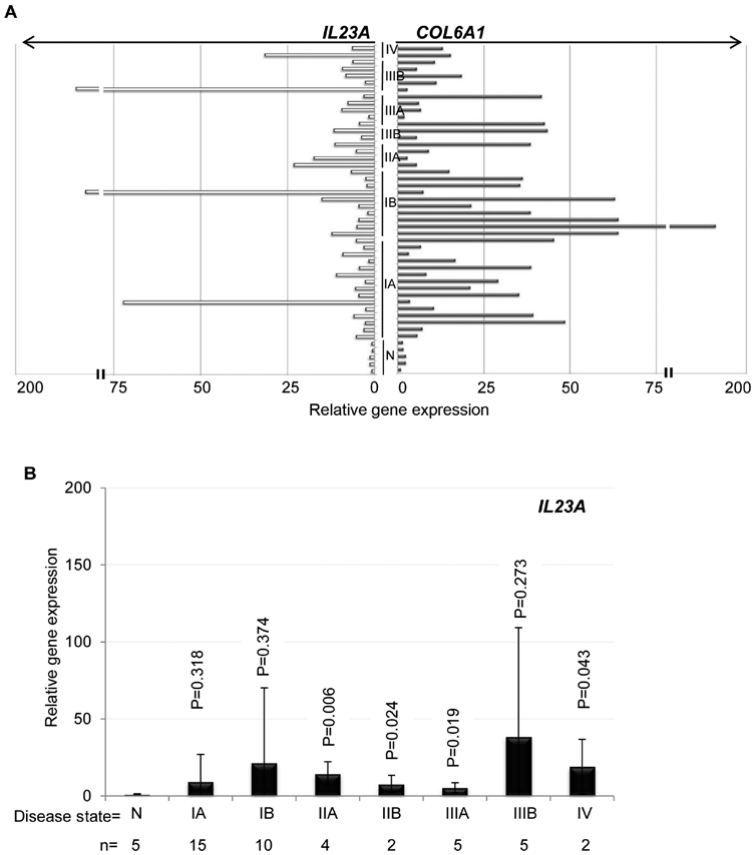
Gene expression of *IL23A* in normal and neoplastic lung tissues. An identical set of TissueScan Lung Cancer Tissue qPCR Panel II (as in Fig. 1) was analyzed for *IL23A* gene expression using TaqMan assay primers with *ACTB* (β-actin) as endogenous control (ABI). (A) Each bar graph represents the expression level of *IL23A* (left panel) or *COL6A1* (right panel) from individual samples covering 4 disease stages (IA, IB, IIA, IIB, IIIA, IIIB and IV) of non-small cell lung cancer and normal tissues (N). (B) The level of *IL23A* expression is presented as mean ± S D in a bar graph from each group. P-value is compared to the normal tissues.

IL-23 has been implicated in the development of cancers (34). The induction of IL-23 by monocytes following COL6 stimulation prompted us to examine whether the levels of IL-23 were elevated in the lung tissues of patients with NSCLC. The expression of *IL23A* encoding the p19 subunit of IL-23 was examined from another duplicate lung cancer tissue panel as in Fig. 1A. Elevated gene expression of *IL23A* was found in lung tissues of NSCLC as compared to that from normal controls (Fig. 3A, left panel). This profile was similar to that of *COL6A1* expression (Fig. 3A, right panel). However, the tissue with higher *COL6A1* expression was not always correlated with higher *IL23A* levels (Fig. 3A). Despite the individual variation and sample sizes, *IL23A* expression was significantly higher at various stages (IIA, IIB, IIIA and IV) of NS CLC than that of normal controls (Fig. 3B).

### COL6 activates the FAK signaling pathway in monocytes

Monocytes express integrins of the β1, β2, β3 and β5 subfamilies (35). Upon binding to its ligands, integrin stimulates the activation of FAK outside-in signaling, which contributes to various biological functions including cytokine production, cell proliferation, survival and differentiation (36). FAK acts as a scaffolding molecule for assembly of the other kinases upon integrin ligation. Activation of FAK by integrin clustering leads to autophosphorylation at tyrosine (Y)397, which is a binding site for the Src homology 2 (SH2) domain of the Src-protein tyrosine kinases. Recruitment of Src family kinases results in the phosphorylation of Y925 in the C-terminal region of FAK (37,38).

**Figure 4:**
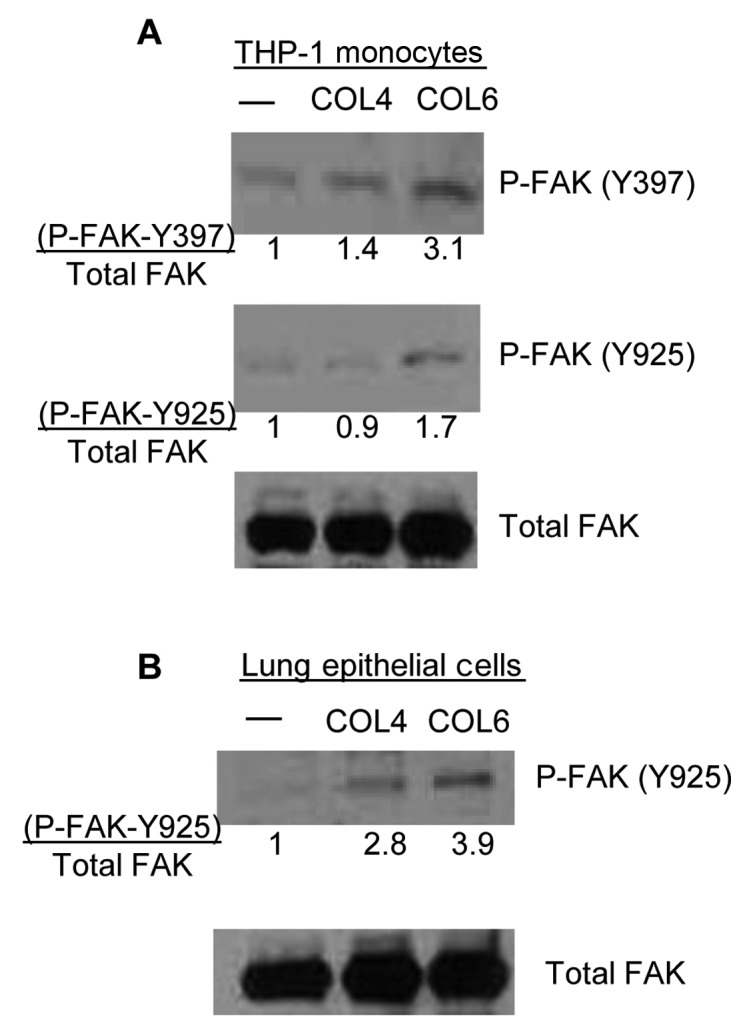
COL6 activates FAK in human THP-1 monocytes and primary epithelial cells. (A) COL6 activates FAK in THP-1 cells. Human THP-1 monocytic cell line was stimulated at 1×10^6^ cells/ml with medium only (−), COL4 (30 μg/ml) and COL6 (30 μg/ml) for 1 h. Activation of FAK was evaluated using western blot analysis with phospho-FAK (P-FAK) at Tyr397 (Y397) and P-FAK (Y925) followed by total FAK antibodies. The band intensity was determined using Image J program and the ratio was calculated from the intensity of P-FAK divided by the intensity of total FAK as shown at the bottom of each blot. (B) COL6 activates FAK in primary epithelial cells. HBEpCs derived from the surface epithelium of normal human bronchi were cultured in Bronchial/Tracheal Epithelial Cell Growth Medium following the manufacturer’s instruction. HBEpCs were stimulated at 1×10^6^ cells/ml with medium only (−), COL4 (30 μg/ml) and COL6 (30 μg/ml) for 1 h. Activation of FAK was evaluated using western blot analysis with P-FAK (Y925) followed by total FAK antibodies. The band intensity was determined using Image J program and the ratio was calculated from the intensity of P-FAK divided by the intensity of total FAK (P-FAK-Y925/Total FAK). Data are representative of similar profiles from two independent experiments using different passages of cells. COL6, type VI collagen; FAK, focal adhension kinase; HBEpCs, human bronchial epithelial cells.

Integrin is one of the receptors for COL6 (21). To test whether the integrin-FAK signaling pathway was activated in monocytes upon COL6 stimulation, human THP-1 monocytes were stimulated followed by analysis of FAK activation using western blot analysis (Fig. 4A). COL4 known to activate FAK in intestinal epithelial cells (39) was included in the stimulation for comparison. We found that FAK was phosphorylated at Y397 and Y925 in THP-1 monocytes stimulated with COL6, suggesting the activation of FAK and Src upon COL6 stimulation. In contrast, COL4 had little if any effects on P-FAK in the monocytes.

### COL6 activates FAK signaling in lung epithelial cells

Signaling through FAK activation has been implicated in the tumorigenic properties of lung cancer cells (40,41). Having shown the activation of FAK in monocytes, we next tested if COL6 was able to stimulate lung epithelial cells that also express integrin. Phosphorylation of FAK at Y925 was not detected from human primary epithelial cells cultured in medium only, while stimulation with COL6 in human primary epithelial cells resulted in an ~4-fold increase in phosphorylation of FAK at Y925 relative to those cultured in medium only. COL4 is known to activate FAK in intestinal epithelial cells (39) and we also observed an intermediate increase (~3-fold) in P-FAK (Y925) upon stimulation with COL4 in lung epithelial cells relative to the control (Fig. 4B).

### COL6 activates MAPK/ERK signaling in lung epithelial cells

**Figure 5:**
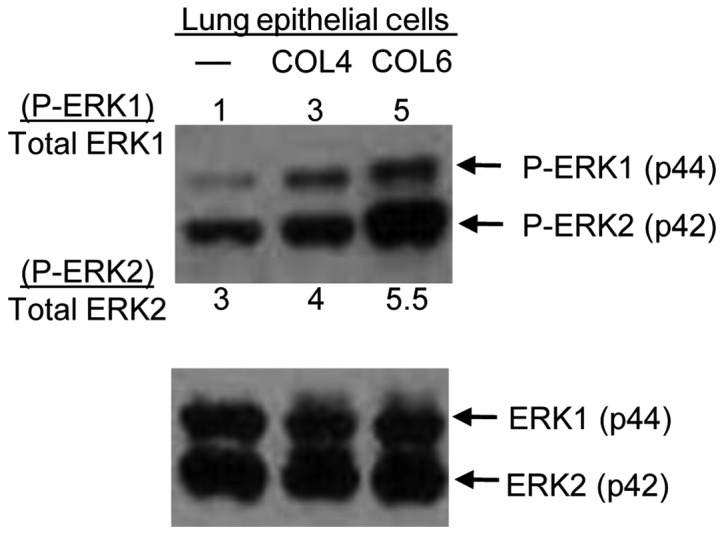
COL6 activates ERK1/2 in human primary epithelial cells. HBEpCs described in Fig. 4B were stimulated with medium only (−), COL4 (30 μg/ml), and COL6 (30 μg/ml) for 1 h. Activation of ERK was evaluated using western blot analysis with phospho-p44/42 ERK1/2 (Thr202/Tyr204) followed by total ERK1/2 antibodies. The band intensity was determined using Image J program and the ratio was calculated from the intensity of P-ERK1 divided by the intensity of total ERK1 (shown on the top of the band) or P-ERK2 divided by total ERK2 (shown on the bottom of the band). Data are representative of similar profiles from two experiments using different passages of primary epithelial cells. COL6, type VI collagen; ERK, extracellular signal-regulated kinase; HBEpCs, human bronchial epithelial cells.

It is known that phosphorylation of FAK at Y925 creates a binding site for the growth factor-receptor-bound protein 2 (GRB2) and activates a small G protein, RAS (37). Activated RAS recruits mitogen-activated protein kinase kinase kinase (MAPKKK or RAF), which leads to activation of mitogen-activated protein kinase kinase (MAPKK or MEK), which then activates the mitogen-activated protein kinase (MAPK) extracellular signal-regulated kinase (ERK)1/2 (42). To further investigate the involvement of ERK1/2 in FAK signaling following COL6 stimulation, we examined the activation of ERK1/2 using western blot analysis. We found that human primary epithelial cells increased the levels of phosphorylation of ERK1/2 upon treatment of COL6 and an intermediate increase in ERK1/2 phosphorylation was also induced by COL4 (Fig. 5).

## Discussion

In the present study, we demonstrated that elevated gene expression of *COL6A1* was present in the lungs with NSCLC at various stages. Lung tissues with adenocarcinoma or squamous cell carcinoma were associated with deposition of more COL6 as compared to normal lungs. COL6 is known for its potent growth-stimulating effects on promoting tumor progression (22,26–29). Our results from *in vitro* cultures suggest a novel function of excess COL6 on stimulating IL-23 production by monocytes. We also found upregulation of *IL23A* expression in neoplastic lung tissues at various stages, consistent with the reports that overexpression of *IL23A* was present in human tumor samples (34). IL-23 has been implicated in the development of cancers by increasing neutrophil and inhibiting CD8^+^ T cell infiltration, thereby promoting tumor incidence and growth (34). Our results suggest a potential role of COL6 in promoting inflammation that is favorable for tumor development.

Although IL-23 was induced by monocytes using a soluble form of COL6 in a dose-dependent manner, it was less efficiently induced using plate-bound COL6 (data not shown). Instead, we found that plate-bound COL6 had effects on endothelial cells, which are also present in the pulmonary interstitium. Primary lung endothelial cells treated with plate-bound COL6 had increased levels of cleaved caspase-3 (data not shown), suggesting the induction of apoptosis. In this study, we did not identify the specific fragments of COL6, which are required for induction of IL-23 expression by monocytes. It is likely that different forms of COL6 are able to stimulate various cell types in physiological conditions.

In order to exhibit stimulatory effects in the lungs, COL6 needs to be secreted and exposed to its surrounding target cells. It is known that COL6 is mainly produced by fibroblasts (43,44) and other cell types such as adipocytes (29) or macrophages are capable of expressing COL6 (45). It has been shown that TGF-β1 and TGF-β3 are responsible for increasing extracellular matrix expression by human lung fibroblasts (46). In addition, COL6 expression is induced by human macrophages following stimulation with IL-4, IL-10, or IL-13, which are involved in T helper type 2 (Th2) allergic inflammation (45). Therefore, the existing inflammatory environment especially mediated by Th2 immunity may increase the expression of COL6 in the lung tissues.

Another possible mechanism for upregulation of *COL6A1* gene expression is due to DNA hypomethylation in stromal cells under hypoxic conditions. It has been shown that a global genomic hypomethylation occurs in synovial fibroblasts from rheumatoid arthritis patients (47). Our preliminary data also found a greatly reduced DNA methylation in the *COL6A1* gene from lung tissue with both emphysema and adenocarcinoma as compared to that of adjacent normal section from the same patient (data not shown). A hypoxic lung environment as in emphysema or lung fibrosis is likely to modulate expression of an array of genes such as ECM component COL6.

To determine if COL6 was increased during the early phases of emphysema, a mouse model of cigarette smoke-induced emphysema was established. Destruction of alveolar septa was evident in mice exposed to cigarette smoke for 4 months (data not shown). However, there was no evidence of increased *Col6a1* transcription or protein deposition in the lung tissues during this phase of cigarette-induced lung injury characterized by lung tissue loss, but no neoplasia (data not shown). These results suggest destruction of ECM alone may not be sufficient to enhance COL6 expression and transform the cells. It is possible this occurs in the faulty repair phase of lung injury, since increased degradation of the ECM leads to aberrant remodeling of the connective tissues and excess matrix protein deposition leads to pulmonary fibrosis, which is sometimes present in the emphysematous lungs. Combined pulmonary fibrosis and emphysema (CPFE) is a recently recognized distinct condition in which the lung is prominently affected by both emphysema and lung fibrosis (48). CPFE may be even more strongly associated with lung cancer than either emphysema or lung fibrosis alone (49). Future investigations will have to mechanistically evaluate whether or not overexpression of COL6 is critical to or it is merely associated with the progression of emphysema or lung fibrosis to lung neoplasm.

In this study, we showed that COL6 exerted its stimulatory effects involving the signaling pathway that activated FAK molecules in monocytes and epithelial cells. MAPK/ERK, the downstream molecule of FAK, was also activated in lung epithelial cells following COL6 stimulation. ERK is known to promote cell proliferation, angiogenesis, cell differentiation and cell survival, which contributes to the development of various types of cancers including NSCLC (31). In addition to promote IL-23-mediated lung inflammation by targeting monocytes, COL6 was capable of activating signaling molecules (e.g., FAK and ERK) in normal lung epithelial cells, which may promote the development of lung cancer. Inhibition of FAK has been beneficial on retardation of tumor progression (38). Studies using small-molecule FAK inhibitor are currently in clinical trials for patients with various types of tumors (50). FAK is a relevant target in the inhibition of tumor progression as well as abrogation of COL6-mediated activity during tissue remodeling.

Destruction of ECM in the lung is regarded as the pathological hallmark of emphysema. Although upregulation of COL6 is important for repairing the wounded tissues during the healing process, excess matrix protein deposition may lead to unwanted consequences, such as increased fibrosis, and/or as we postulate, increased tumorigenesis potential. In this report, we defined that COL6 exerted stimulatory effects on inducing IL-23 production by monocytes, supporting its role in activating immune cells to promote pro-tumor lung inflammation during the process of tissue remodeling. Amelioration of the progression or abnormal reparative phases of emphysema or lung fibrosis by blocking signaling pathways exerted by excessive ECM components such as COL6, may be an important step in inhibiting tumorigenesis.
